# Reliability, minimal detectable change and responsiveness to change: Indicators to select the best method to measure sedentary behaviour in older adults in different study designs

**DOI:** 10.1371/journal.pone.0195424

**Published:** 2018-04-12

**Authors:** Manon L. Dontje, Philippa M. Dall, Dawn A. Skelton, Jason M. R. Gill, Sebastien F. M. Chastin, on behalf of the Seniors USP Team

**Affiliations:** 1 School of Health and life Science, Institute of Applied Health Research, Glasgow Caledonian University, Glasgow, United Kingdom; 2 Institute of Cardiovascular and Medical Sciences, University of Glasgow, Glasgow, United Kingdom; 3 Department of Movement and Sports Sciences, Ghent University, Ghent, Belgium; Sao Paulo State University - UNESP, BRAZIL

## Abstract

**Introduction:**

Prolonged sedentary behaviour (SB) is associated with poor health. It is unclear which SB measure is most appropriate for interventions and population surveillance to measure and interpret change in behaviour in older adults. The aims of this study: to examine the relative and absolute reliability, Minimal Detectable Change (MDC) and responsiveness to change of subjective and objective methods of measuring SB in older adults and give recommendations of use for different study designs.

**Methods:**

SB of 18 older adults (aged 71 (IQR 7) years) was assessed using a systematic set of six subjective tools, derived from the TAxonomy of Self report Sedentary behaviour Tools (TASST), and one objective tool (activPAL3c), over 14 days. Relative reliability (Intra Class Correlation coefficients-ICC), absolute reliability (SEM), MDC, and the relative responsiveness (Cohen’s d effect size (ES) and Guyatt’s Responsiveness coefficient (GR)) were calculated for each of the different tools and ranked for different study designs.

**Results:**

ICC ranged from 0.414 to 0.946, SEM from 36.03 to 137.01 min, MDC from 1.66 to 8.42 hours, ES from 0.017 to 0.259 and GR from 0.024 to 0.485. Objective average day per week measurement ranked as most responsive in a clinical practice setting, whereas a one day measurement ranked highest in quasi-experimental, longitudinal and controlled trial study designs. TV viewing–Previous Week Recall (PWR) ranked as most responsive subjective measure in all study designs.

**Conclusions:**

The reliability, Minimal Detectable Change and responsiveness to change of subjective and objective methods of measuring SB is context dependent. Although TV viewing-PWR is the more reliable and responsive subjective method in most situations, it may have limitations as a reliable measure of total SB. Results of this study can be used to guide choice of tools for detecting change in sedentary behaviour in older adults in the contexts of population surveillance, intervention evaluation and individual care.

## Introduction

Prolonged sedentary behaviour is common at all ages and is associated with a range of health problems [1]. National physical activity guidelines have started to acknowledge this with recommendations to reduce prolonged sedentary behaviour [2,3]. As a result, several countries are now including monitoring of sedentary behaviour within their surveillance systems and an increasing number of studies are evaluating interventions aimed at reducing sedentary behaviour [4–6]. To assess and interpret changes in sedentary behaviour, over time or as a result of an intervention, measures that are reliable and responsive to change are vital.

Sedentary behaviour can be measured subjectively with surveys, questionnaires,or other self-reported methods. It can also be measured objectively, for example with accelerometers. Both methods have specific pros and cons for use, but the vast majority of the literature on the measurement characteristics of sedentary behaviour measures is focused on their validity. Some studies have examined how their reliability affects their ability to obtain accurate estimates of sedentary behaviour in specific samples and populations, but not how it affects their ability of detecting and measuring change [7,8]. Currently there is a real dearth of information to guide choice of measurement tools for the purpose of measuring and detecting change in sedentary behaviour [9].

A change in sedentary behaviour can be interpreted as a true change in behaviour only if the observed difference between two measurements is larger than the measurement error. The measurement error is a combination of two elements: (1) random error intrinsic to the measurement tool and measurement procedure used (e.g. variability in recollected sedentary behaviour between and within individuals for a self-reported tool or variation in wear time of an objective measure); and (2) natural variability in behaviour (e.g. day to day variation in behaviour or seasonal variations will affect total sedentary behaviour [10]).

How these two elements combine into a measurement error impact the ability of a measurement tool to measure and detect change, depends on the context in which it is used, as highlighted by Beaton’s taxonomy of responsiveness [11]. There are several statistical indicators (e.g. Intraclass Correlation Coefficient (ICC), Minimal Detectable Change (MDC), Cohen’s d effect size (ES) or Guyatt’s Responsiveness (GR) coefficient) that can be used to evaluate this. Which index measure is appropriate is depends on:

*Whose change needs to be measured and detected*: is it the change in behaviour of a single individual (typical for a clinical context) or the change of a group (typical for surveillance studies)?
*Which data are being compared*: are the scores being contrasted over time (within-subject study design—typically a longitudinal study or a quasi experimental trial) or at one point in time (between-subject study design—typical of intervention studies involving a control group)?
*What type of change is being quantified*: is the type of change being quantified the observed change or the clinically relevant change?

To date, information to guide choice of measurement tools for the purpose of measuring and detecting change in sedentary behaviour in the contexts of population surveillance, intervention evaluation and individual care is lacking. Therefore, the aim of this study was to provide systematic comparative information about the reliability, minimal detectable change, and responsiveness to change for objective measures and a representative selection of currently available self-reported tools (including previous day recall vs previous week recall, total sedentary time vs proxy measures vs composite measures) for the different study designs.

## Material and methods

### Study design and study sample

This study is based on a repeated-measure design, during which the sedentary behaviour of older adults was measured for a period of two consecutive weeks (14 consecutive days) using objective and subjective measures. This number of measurement days was necessary to capture natural day-to-day variability and intrinsic measurement random error. To clarify, individual measurements of sedentary behaviour may vary due to day-to-day variability as well as due to measurement error related to the type of measurement. When sedentary behaviour is only measured on two days, the natural differences in daily activities (eg, playing cards on Tuesdays versus playing golf on Fridays) might result in biased conclusions about the effect of interventions or time. A measurement period of 14 days allowed the capture of natural day-to-day variability more extensively. In addition, by measuring 14 days it was possible to examine and compare the intrinsic measurement error of the most common types of self-reported and objective measures of sedentary behaviour in one study (ie, previous day recall measurements, previous week recall measurements, 1 day objective measurements, average day objective measurements).

Participants were recruited from the Glasgow Caledonian University Older Adult Research Database. This database contained 99 individuals (as of May 2016) with a variety of controlled medical conditions, all of whom have previously consented to be contacted by academic researchers concerning potential participation in research projects. The inclusion criteria were: 65+ years of age, community dwelling and able to ambulate independently. Potential participants were excluded when they lacked capacity to consent, to use the equipment, or to complete the questionnaires. This study received ethical approval from Glasgow Caledonian University School of Health and Life Sciences Ethics Committee. The study complied with the principles outlined in the Declaration of Helsinki. All participants gave their written informed consent.

### Procedure

**Fig 1:**
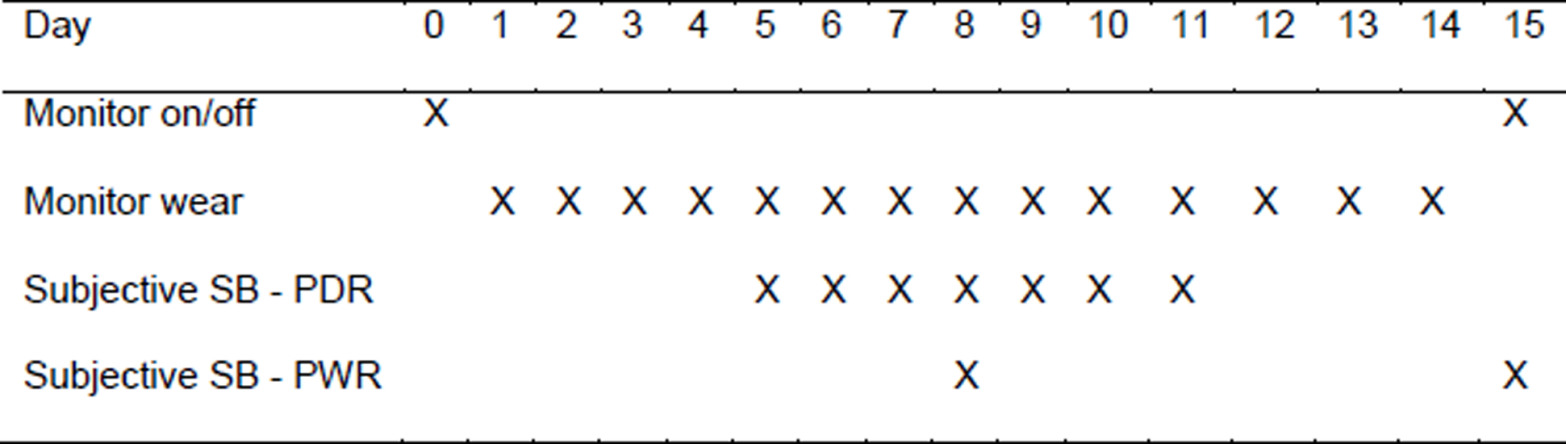
Time-points of objective and subjective measurements (SB = sedentary behaviour, PDR = previous day recall, PWR = previous week recall).

Fig 1 details the time points of all measures recorded. One day prior to the measurement eligible participants were visited at home by a researcher. During this first visit participants gave their written informed consent, baseline demographic information was collected, and the researcher attached the activity monitor (Activpal3c). The monitor was attached to the anterior thigh of the dominant leg of the participant with a waterproof dressing. The monitor was programmed to start data collection at midnight of that day, and was worn continuously for the following 14 full days. While wearing the activPAL3 participants were asked to continue their normal daily activities. Since the activPAL3 monitor cannot make a distinction between sitting/lying while sleeping or while awake, participants were asked to note the time they fell asleep the previous night and the time they woke up every day in a sleep diary. On seven days (Day 5 to Day 11) they were asked to complete previous day recall questionnaires about their time spend sedentary, and on two days (Day 8 and Day 15) they were asked to self-report time spend sedentary in the previous week. On Day 15 the researcher removed the activity monitor and collected the sleep diary and questionnaires.

### Measurements

#### Objective measurement of sedentary behaviour

Sedentary behaviour (SB) was objectively measured with an activity monitor (activPAL3 physical activity monitor, PAL Technologies Ltd, Glasgow, Scotland). The activPAL3 is a small and user-friendly tri-axial activity monitor, frequently used as gold standard for objective measurement of sedentary time and pattern [12–15]. Outputs include time spent sitting/lying, standing, and stepping, number of steps and number of sit-to-stand transitions. The monitor was heat-sealed inside a plastic pouch to make it waterproof, and was therefore worn continuously for the entire 14 days of data collection.

#### Subjective measurement of sedentary behaviour

**Fig 2:**
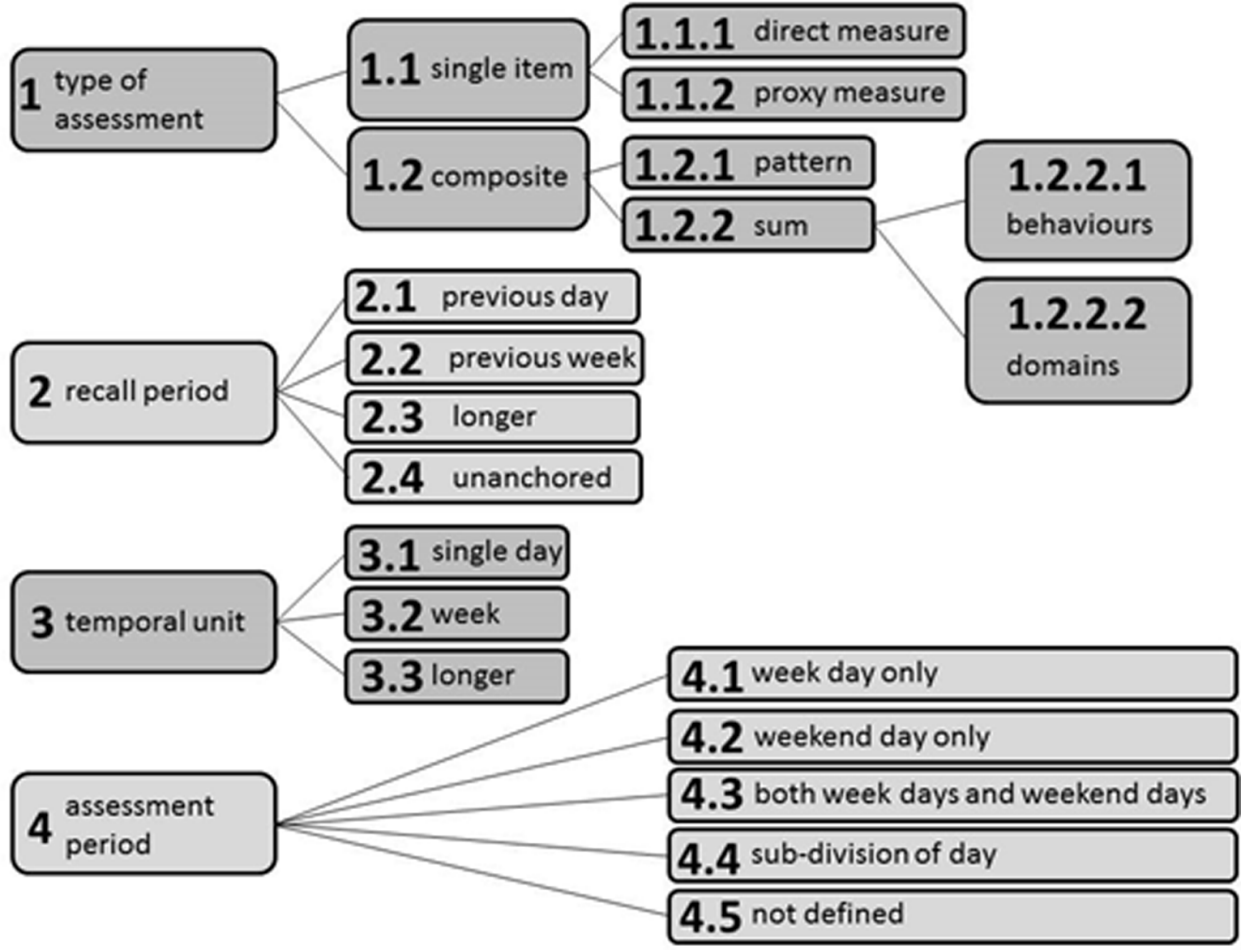
TASST framework. Reproduced from Fig 1 Dall PM, Coulter EH, Fitzsimons CF, Skelton DA, Chastin SFM, on behalf of the Seniors USP Team. The TAxonomy of Self-reported Sedentary behaviour Tools (TASST) framework for development, comparison and evaluation of self-report tools: content analysis and systematic review. BMJ Open 2017;7:e013844 [9].

To provide a systematic comparison between existing sedentary behaviour self-report measurement tools participants also completed a sedentary behaviour questionnaire (covering three types of assessment), based on the TAxonomy of Self-report SB Tools (TASST) framework (Fig 2) [9]. The TASST framework can be used to describe most variations of currently available sedentary behaviour self-report measurement tools. The TASST framework consists of four domains, which show different characteristics of measurement tools: type of assessment, recall period, temporal unit and assessment period. Using the framework as a tree, a single end point (taxon) can be identified to describe specific questions in a tool. The SB questionnaires used for this study are available in the Supplementary Material (S1 Appendix. Daily SB questionnaire) and were developed for use in the Seniors USP: Understanding Sedentary Patterns Study (see acknowledgments).

Two recall time periods, i.e. previous day (TASST taxon 2.1) and previous week (7 day recall) (TASST taxon 2.2) were reported in the questionnaire. In both cases the temporal unit was a day (TASST taxon 3.1; i.e. all questions asked about time spent sitting per day), and the assessment period was not defined (TASST 4.5; i.e. week or weekend days were not considered separately). Within each set of questions, participants were asked to report total time spent sitting (TASST taxon 1.1.1) and also the time spent sitting while watching TV, using a computer, reading, listening to music, doing a hobby, talking with friends or family, eating, performing self-care tasks, doing household tasks, and taking a nap during the day or resting while doing nothing else. The time spent watching TV was used as a proxy measure of sitting (TASST 1.1.2), and all ten behaviours were added together to form the composite sum of time in different SBs (TASST taxon 1.2.2.1). Therefore, in total six different self-report tools were assessed; three types (total sitting, a TV proxy measure, and composite sum of time in different SBs) in each of two recall periods (previous day and previous week) (see S1 Appendix. Daily SB questionnaire for the complete questionnaire).

### ActivPAL data processing

The statistical programming environment and language R [16] was used to process sleep diary data with activPAL3 data (event output downloaded using activPAL software version 7.2.32, PAL Technologies Ltd, UK) to calculate total time spent sedentary, time spent standing, time spent walking, number of steps and number of sit-to-stand transitions during waking hours for each 24 hour period, each beginning at midnight. The outcome measures for this study were, the total time spent sedentary on each day during waking hours, and for both weeks, the average time spent sedentary per day (as calculated by dividing the sum of the total time spent sedentary per week by 7).

### Statistical indicators

A frequently used method to examine the measurement error is a reliability measure, i.e. to examine the consistency between scores. Reliability, as in consistency, can be divided into relative reliability and absolute reliability. Relative reliability is about the consistency of the position or rank of individuals in the group relative to other, and absolute reliability is about the consistency of the scores of individuals [17]. The Intraclass Correlation Coefficient (ICC) is often used as a measure of relative reliability. The ICC gives the ratio of variances due to differences between subjects [17]. However, a high ICC, indicating a high *relative* reliability, does not give an indication of the accuracy of individual measurements [18]. The Standard Error of Measurement (SEM) quantifies the precision of the individual measurements and gives an indication of the *absolute* reliability [17–19]. The SEM can be used to calculate the Minimal Detectable Change (MDC), which is the minimal amount of change that a measurement must show to be greater than the within subject variability and measurement error, also referred to as the sensitivity to change [17,18,20]. The measurement error also affects the responsiveness of the measure, which is the ability of the measure to detect any change [21] and is often expressed by a statistical coefficient (dividing difference in mean by a measure of variability), such as Cohen’s d effect size (ES) or Guyatt’s Responsiveness coefficient (GR) [11,21,22].

### Statistical analyses

Descriptive statistics were used to describe the study sample. Scatterplots were created for each outcome measure to visually present the daily variation in sedentary behaviour. Checking the outcome measures revealed two extreme outliers in previous week recall of total sitting time, and three in the proxy measures in the previous week recall. It was clear that the participants misinterpreted the questions; for example when reporting more than 24 hours of sedentary time per day it was clear that they estimated the total time per week instead of the time on an average day in the previous week. Therefore, those reported values were divided by 7 to get a value for an average day value instead of a total value for a week.

To examine the relative and absolute reliability of each tool, we used a 3-layered approach as recommended by Weir et al. [17].

1. First, a single-factor, within-subjects repeated-measures ANOVA was conducted for each outcome measure. In detail: with the single day objective outcome, the repeated-measures ANOVA examined if there was a difference between the 14 days; with the previous day subjective outcome if there was a difference between the 7 days; with the previous week recall and the average objective day, if there was a difference between 2 measurements. The inferential test of mean differences (F-test) was evaluated to check if there was a systematic error between the days. Because there was no change induced, no systematic difference between the days was expected, despite some variability between the days. The partial eta squared (η_p_^2^) was calculated to show the effect size of the ANOVA.
2. Second, Intra Class Correlation coefficients (ICC_2,1_ for daily objective measurements and subjective measurements, or ICC_2,2_ for average day objective measurement) were calculated to examine the relative reliability [18]. A higher ICC represents a more favourable relative reliability.
3. And third, the error term from the 2 way model (MS_E_) of the repeated measures ANOVA was used to calculate the Standard Error of Measurement (SEM) (SEM = square root of the mean square error term from the ANOVA) [17], which represents the absolute reliability. A smaller SEM indicates a better absolute reliability of the measure.

The SEM was used to calculate the Minimal Detectable Change (MDC) of each tool with the following formula: MDC = Standard Error of Measurement X 1.96 X √2 [20]. The MDC indicates the minimal amount of change that can be interpreted as a real change in sedentary behaviour for an individual; a smaller MDC indicates a more sensitive measure [17,18,20].

In addition, Cohen’s effect size (ES) and Guyatt’s responsiveness coefficient (GR) were calculated for each measure [21]. To calculate ES and GR, two measurements of each measure were needed. For the previous week recall measurements there were two measurements available in this study. This also applies for the average day objective measurements. For the previous day recall, there were seven measurements available, so Day 5 was selected as measurement 1 and Day 11 as measurement 2 to calculate ES and GR. For the daily objective measurement there were 14 measurement days available, and Day 5 and Day 11 were selected for comparability with the self-report measures. Cohen’s effect size was calculated by dividing the difference between the mean of measurements 1 and 2 by the standard deviation of measurement 1 (Δ/SD). Guyatt’s responsiveness coefficient was calculated by dividing the difference between the mean of measurements 1 and 2 by the SEM (Δ/SEM). Cohen’s effect sizes and Guyatt responsiveness coefficients are usually interpreted such that values of 0.2, 0.5 and 0.8 represent small, moderate and large responsiveness [23–25]. In the present study, however, the responsiveness coefficients can only be interpreted in a relative manner to each other, because no change was induced. The coefficients can be compared to assess which measures are more responsive than others [25]. Larger values of Cohen’s effect size and Guyatt responsiveness coefficients represent a more responsive measure.

The SEM and MDC can be used to interpret the individual changes in behaviour, whereas the responsiveness coefficients can be used to evaluate changes at group level [17,25].

To examine which method is most responsive to change, Beaton’s responsiveness taxonomy was used to rank the methods for a specific context [11] (Table 1). Three Beaton taxa were assessed, representing commonly used study designs. The first Beaton taxon used was a clinical setting, where the results are presented for an individual (Who), where within-subject scores are being contrasted over time (Which), and the type of change being quantified is indicated by the Minimal Detectable Change (What). The second Beaton taxon used was quasi-experimental or longitudinal study design, where the results are presented for a group (Who), where within-subject scores are being contrasted over time (Which), and the type of change being quantified is the observed change in the population indicated by the ES (What). The third Beaton taxon used was based on study design of a controlled trial, where the results are presented for a group (Who), where between-subject scores are being contrasted over time (Which), and the type of change being quantified is the observed change in the population indicated by the GR (What).

**Table 1:** Ranking of methods to measure sedentary behaviour based on responsiveness in different contexts, as classified in Beaton’s responsiveness taxonomy [11].

Taxon of Beaton	WHO	WHICH	WHAT	Indicator	In which context is this indicator applicable?	Ranking of objective measurement methods *(from most responsive to least responsive)*	Ranking of subjective measurement methods *(from most responsive to least responsive)*
1.2.2	Individual	Within-subject change over time	Minimal detectable change	MDC	Clinical practice	1. Average day per week 2. One day	1. TV viewing—PWR 2. TV viewing—PDR 3. Total SB—PWR 4. Total SB—PDR 5. CSB—PDR 6. CSB—PWR
2.1.3	Group	Within-subject change over time	Observed change in population	ES	Quasi-experimental or longitudinal study	1. One day 2. Average day per week	1. TV viewing—PWR 2. CSB—PDR 3. Total SB—PDR 4. CSB—PWR 5/6. Total SB—PWR / TV viewing–PDR
3.3.3	Group	Between- subject change over time	Observed change in population	GR	Controlled trials	1. One day 2. Average day per week	1. TV viewing—PWR 2. CSB—PDR 3. CSB—PWR 4. TV viewing—PDR 5. Total SB—PDR 6. Total SB–PWR

MDC = Minimal Detectable Change; ES = Cohen’s d effect size; GR = Guyatt responsiveness coefficient; CSB—composite sum of time in different SBs; PWR = previous week recall; PDR = previous day recall; SB = sedentary behaviour

## Results

### Study sample

Twenty-two participants were recruited for this study. Three participants had insufficient activPAL3 data (<14 days) and one participant had a faulty activPAL3 measurement, and were excluded from the analysis sample. The median (IQR) age of the analysis sample (N = 18) was 71 (7) years and 72% were men. The mean (SD) body mass index was 25.43 (3.05) kg/m^2^.

### Objectively measured sedentary behaviour

**Fig 3:**
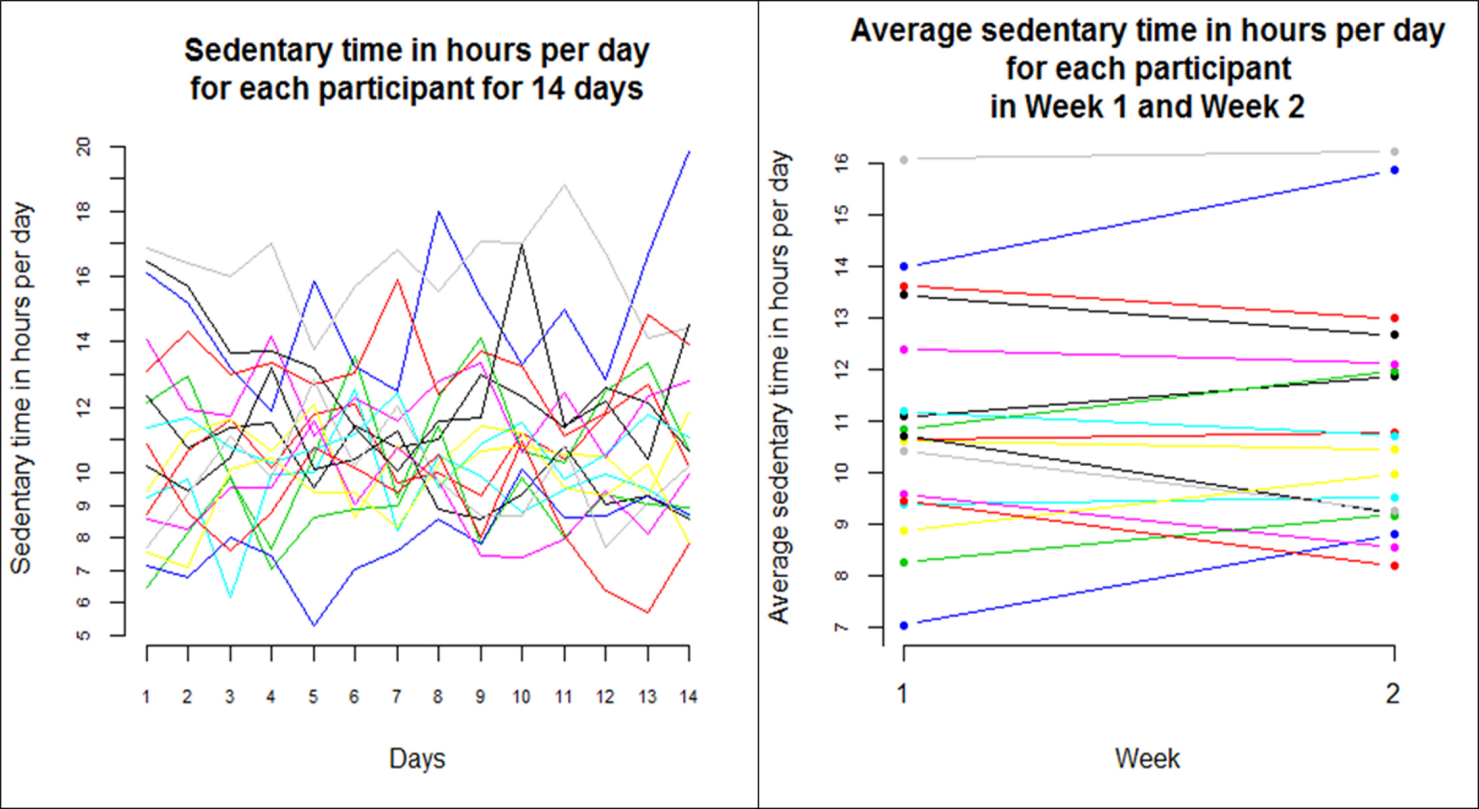
Variation in objectively measured sedentary behaviour. Left: Daily sedentary behaviour for a period of 14 days (N = 18). Right: Average daily sedentary behaviour in Week 1 and Week 2 (N = 18).

Based on the objective measurements, the mean (SD) time spent sedentary per day was 11.01 (2.22) hours. The variation in daily sedentary time was large, as presented in Fig 3. The day-to-day variation in one day measurements (Fig 3, left) was considerably larger than the variation in the average time spent sitting per day in Week 1 and Week 2 (Fig 3, right). The differences between the days (F = 0.271, p = 0.995) and between the averages per week (F = 0.024, p = 0.879) were not significant, so even though sedentary behaviour varied between days, it was not a systematic trend (Table 2). For both the relative and absolute reliability, the average day per week measurements (ICC_2,2_ 0.946, SEM 43.85 min) were better than the one day measurements (ICC_2,1_ 0.693, SEM 87.46 min). The Minimal Detectable Change was also better (smaller) for average day per week measurements (2.03 hrs) than for one day measurements (4.04 hrs), but the responsiveness coefficients were better (larger) for the one day measurements (Table 2).

**Table 2:** Relative reliability, absolute reliability, Minimal Detectable change and responsiveness of tools to measure sedentary behaviour (N = 18).

	Taxon of TASST	Baseline	Post	Systematic difference	ICC	SEM	MDC	ES	GR
		mean (SD) in min	mean (SD) in min		(95% CI)	in min	in hours		
**OBJECTIVE MEASUREMENTS** (ACTIVPAL)									
1 day^a^		665.70	648.35	F(13,221) = 0.271,	0.693	87.46	4.04	0.126	0.140
		(137.56)	(158.10)	p = 0.995, η_p_^2^ = 0.016	(0.544–0.84)				
Average day per week^b^		659.50	661.76	F(1,17) = 0.024,	0.946	43.85	2.03	0.017	0.036
		(134.01)	(139.91)	p = 0.879, η_p_^2^ = 0.001	(0.856–0.98)				
**SUBJECTIVE MEASUREMENTS**									
Previous day recall^c^									
Total SB	1.1.1/2.1/3.1/4.3	479.41	482.94	F(6,96) = 0.668,	0.414	99.59	4.60	0.037	0.025
		(96.65)	(150.12)	p = 0.676, η_p_^2^ = 0.040	(0.227–0.655)				
TV viewing	1.1.2/2.1/3.1/4.3	194.17	195.83	F(6,102) = 1.366,	0.595	68.08	3.15	0.019	0.033
		(104.49)	(108.85)	p = 0.236, η_p_^2^ = 0.074	(0.412–0.783)				
Composite sum of time in different SBs	1.2.2.1/2.1/3.1/4.3	598.61 (162.42)	631.39 (323.25)	F(6,102) = 1.059, p = 0.392, η_p_^2^ = 0.059	0.743 (0.591–0.874)	137.01	6.33	0.202	0.169
Previous week recall^d^									
Total SB	1.1.1/2.2/3.1/4.3	496.23	493.06	F(1,17) = 0.01,	0.531	93.43	4.30	0.019	0.024
		(163.06)	(103.14)	p = 0.92, η_p_^2^ = 0.0006	(0.1–0.794)				
TV viewing	1.1.2/2.2/3.1/4.3	188.94	213.67	F(1,17) = 4.238,	0.856	36.03	1.66	0.259	0.485
		(95.33)	(94.65)	p = 0.055, η_p_^2^ = 0.200	(0.657–0.944)				
Composite sum of time in different SBs	1.2.2.1/2.2/3.1/4.3	693.11 (468.11)	681.39 (236.41)	F(1,17) = 0.037, p = 0.849, η_p_^2^ = 0.002	0.758 (0.462–0.902)	182.4	8.42	0.025	0.045

ICC = Intraclass Correlation Coefficient; TASST = Taxonomy of self-report SB tools framework [9] SEM = Standard Error of Measurement; MDC = Minimal Detectable Change; ES = Cohen’s effect size (Δ/SD); GR = Guyatt’s responsiveness coefficient (Δ/SEM), SB = sedentary behaviour.

Measurement days used in all analyses except for ES and GR: a) Day1-Day14; b) average Day1-7 and average Day8-15; c) Day5-Day11; d) Day8 and Day15

Measurement days used for ES/GR analyses: Day5 and Day11 (average Day1-7 and average Day8-15 was used for ES/GR analyses in Objective–Average per week)

### Subjectively measured sedentary behaviour

**Fig 4:**
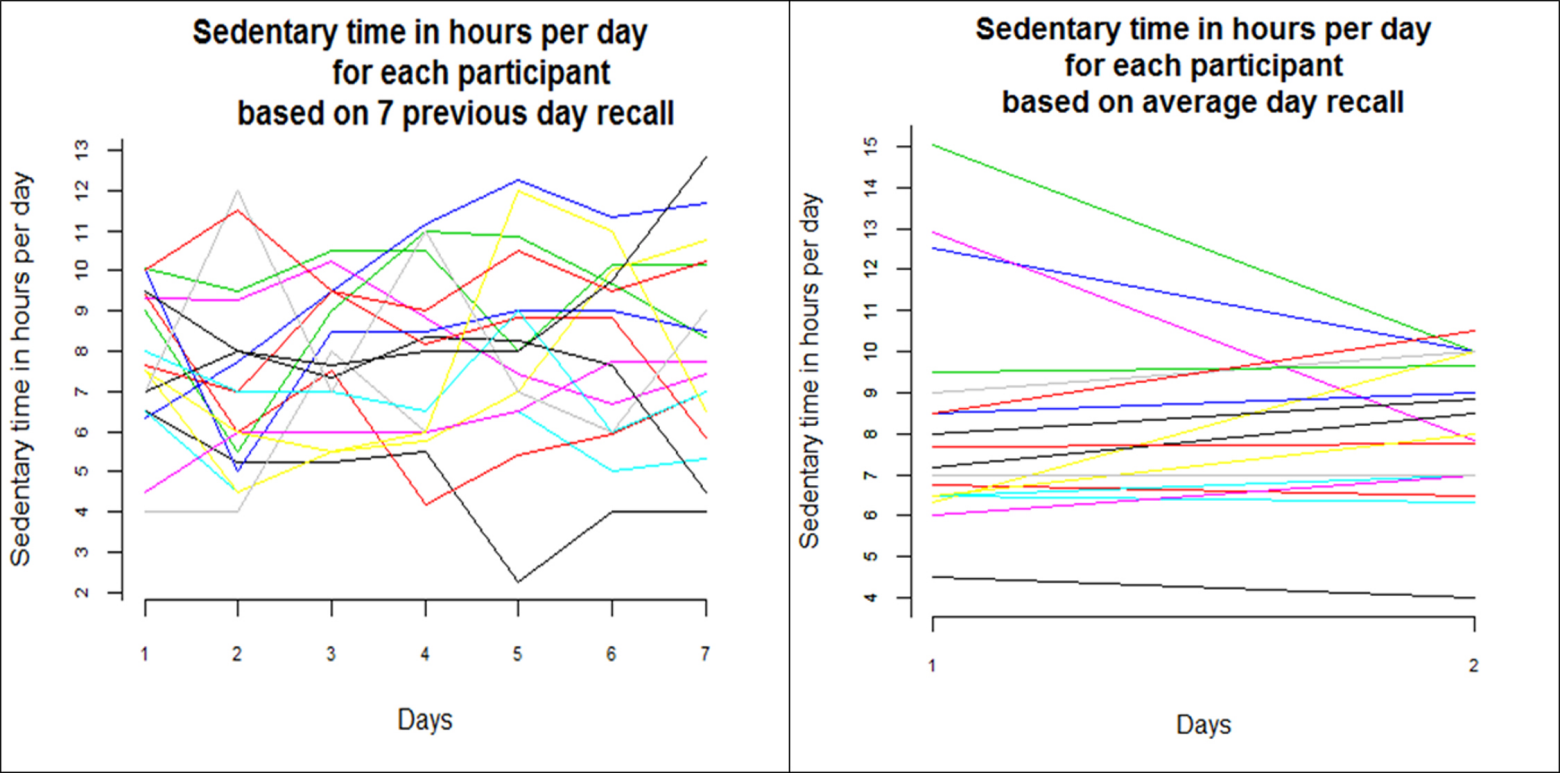
Variation in subjectively measured total sedentary behaviour. Left: Total daily sedentary behaviour measured with a previous day recall questionnaire on 7 days (N = 18). Right: Average total sedentary behaviour per day in Week 1 and Week 2 based on a previous week recall questionnaire (N = 18).

Based on subjective previous day recalls, participants felt they were sedentary on average 7.80 (SD 1.53) hours per day, which was considerably less than the objective measures recorded. The previous day recall measurements captured more individual (within subject) variation in total sedentary time than the previous week recall measurements (Fig 4). Although there were differences between the days, these were non-significant (Table 2). This followed expectations, since there was no change in behaviour induced. Both the relative and absolute variability as well as the MDC were slightly more favourable for total SB measured with previous week recall than measured with the previous day recall, but the responsiveness coefficients were better (larger) for previous day recall (Table 2).

The relative and absolute reliability (ICC and SEM), the MDC and the ES/GR responsiveness coefficients of the other subjective measurements are presented in Table 2. A number of comparisons can be drawn.

Within the same recall period (previous day or previous week), total sedentary behaviour measures showed better absolute reliability (SEM) and smaller MDC, but worse relative reliability and responsiveness, than the sum of behaviours. The absolute and relative reliability, MDC and most responsiveness coefficients of TV viewing were better than the total sedentary behaviour measures within the same recall period. In both recall periods, the absolute and relative reliability and the MDC were better for TV viewing than the sum of behaviours. With a previous week recall period, TV viewing was also more responsive to change than sum of behaviours, but the opposite was true with a previous day recall period.

Comparing types of assessments across the different recall periods, all measures of relative reliability (ICC) are worse for the previous day recall than the previous week recall. For absolute reliability and minimal detectable change, total sitting and TV viewing were better for previous day recall, but sum of behaviour is worse. However, the effect of recall period on responsiveness was not clear-cut: previous day recall was better for both indices for the sum of behaviours, and worse for both indices for TV viewing, but was better for the ES and almost equal for the GR for total sitting.

### Which method is most responsive to change?

Table 2 gives detailed information regarding the absolute and relative reliability and of the responsiveness to change of the different methods to measure sedentary behaviour. Table 1 shows how the methods are ranked from most responsive to least responsive within specific contexts as specified by the responsiveness taxonomy of Beaton [11].

For example, within a clinical setting the most responsive objective method is measuring the average day per week (MDC = 2.03 hours). The most responsive subjective method is asking about the time spent watching TV on an average day in the previous week (MDC = 1.66 hours) and the least responsive subjective method is the composite sum of time in different SBs on an average day in previous week recall questionnaire (MDC = 8.42 hours).

Within a quasi-experimental or longitudinal study setting, the most responsive objective method is a one day measurement (ES = 0.126). The most responsive subjective method is asking about the time spent watching TV on an average day in the previous week (ES = 0.259), whereas the least responsive method is asking about the total time spent sedentary on an average day in the previous week (ES = 0.019) or asking about TV viewing on the previous day (ES = 0.019).

In a controlled trial setting, the most responsive objective method is a one day measurement (GR = 0.140). The most responsive subjective method is asking about the time spent watching TV on an average day in the previous week (GR = 0.485), and the least responsive subjective method is asking about the total time spent sedentary on an average day in the previous week (GR = 0.024).

## Discussion

In this study several statistical indicators were examined to provide systematic comparative information about the most appropriate tool to measure and interpret change in sedentary behaviour in older adults. The results show that there is no one tool that is the best in every situation, but that the choice of tool is context dependent. Indeed a recent article by Kelly at al (2016) also suggested that the context is key in the choice of measurement tool [26] and a careful balance between accuracy, reliability and responsiveness needs to be achieved that suits the specific aim and design of the study or surveillance.

Overall, for objective measurements, a seven day monitoring period provides a more stable measure then a one day measurement period (ICC = 0.946 vs ICC = 0.693, respectively) with better absolute reliability (SEM = 43.85min vs SEM = 87.46min), as previously reported in several studies [8,27,28]. Also consistent with previous results, is the fact that reliability degrades if the monitoring period is shortened. In contrast, however, responsiveness to change improves with a shorter recording period. In intervention or longitudinal studies, where responsiveness is important, shorter assessment periods could be considered. Although this may have consequences for reliability and further research is warranted, it would lessen both research cost and participant burden considerably, and potentially allow the more widespread use of objective measures. However, although an objective measure was always amongst the top three performing measurements for all of the evaluated indicators, objective measures did not consistently outperform subjective ones on all statistical indicators. Objective monitors are acknowledged to be more accurate than subjective ones, as also indicated by the large difference in subjective and objective sedentary time in this study. However, if absolute accuracy is not a major consideration in a study, then one could consider opting for a carefully chosen subjective tool from the TASTT taxonomy for larger scale surveillance.

For studies that cannot afford (in cost or participant burden) objective measures, the choice of self-reported tool needs to be guided by the primary aim of the study and its design. Overall TV viewing time appears to have the best measurement characteristics in most cases. However this is probably due the fact that there is both less natural variability in TV viewing time and less difficulty in recalling it, resulting in less random error. However, care should be taken in choosing TV viewing as a self-reported measure to assess change in sedentary behaviour more broadly, as it is a proxy measure assessing only one component of total sitting time. This has two major implications for its use as an outcome measure. Firstly, the low absolute value for MDC for TV viewing (in hours), is likely to be a reflection of the fact that TV viewing represents a smaller amount of total sitting time, and it may represent a large percentage change in TV viewing. Secondly, while TV time and self-reported sedentary time are correlated, a change in TV time does not guarantee a change in total sedentary time. In fact, the high relative reliability (ICC) reported for the composite sum of time in different SBs suggests that there may be a lot of natural transfer of time between sedentary behaviours e.g. swapping TV watching for reading. In consequence, in an intervention to reduce total sedentary behaviour, it is not clear that a reduction in time spent watching TV would actually represent a reduction in total sitting time. Taken together, this has a few implications for future research. If interested in assessing change in total sitting time, using TV viewing as a proxy measure is not advisable. However, for studies that are interested in assessing a change in TV time, previous week TV viewing is a very good measure to use.

For studies interested in total sedentary time, it is possible to make the following recommendations. Relative reliability (ICC) was better for the sum of behaviours (TASST taxon 1.2.2.1) than a single item direct measure of sitting for both previous day (TASST taxon 2.1) and previous week (TASST taxon 2.2) recall periods. If the aim of the measurement is to examine change of an individual subject over time (MDC), then the previous week recall of a single item direct measure is preferable ((TASST taxon 1.1.1/2.2). However, for quasi-experimental, longitudinal, and RCT studies (GR and ES), a previous day recall of the sum of behaviours is preferable (TASST taxon 1.2.2.1/2.1).

Strenghts of this study included the longer measurement period that allowed capturing day-to-day variability in sedentary behaviour as well as intrinsic measurement random error of a number of self-reported and objective measurements; rigorous testing and detailed consideration of measurement proporties; and usability of the findings by providing a ranking for the most common measurements and study designs. However, when interpreting the results of this study a few limitations need to be considered, such as the small sample size, and the limited generalisibility of the findings. First, although the study sample size might seem small, the statistical indicators seemed largely independent of sample size. For example, literature shows that the SEM is a fixed characteristic of the measure, independent of the study sample [29,30]. However, extreme scores within a study sample might affect the SEM [29]. Therefore, we repeated the same analyses with two random subsamples (N = 10). All indicators were similar (results not shown) which confirmed the stability of the indicators across samples. Secondly, care should be taken when interpreting the large absolute values of Minimal Detectable Change reported in this study. MDC is a metric reporting the minimal amount of change that a measurement must show to be certain that it is a true change, i.e. beyond variability and measurement errors [20,22,31,32]. However, this only applies to a change for an individual, and is thus only relevant when assessing a change in an individual in a clinical situation. Whether such large changes in individuals are realistic is unknown. A longer measurement period might be necessary to improve the sensitivity to change of the specific measurements, but more research is necessary to confirm this. When assessing the change in groups means, for example in a randomised controlled trial, the difference required could be lower, however due to the design of this study we did not have the data to evaluate the potential size of this value. While the Minimal Detectable Changes of all measures seem relatively large, that does not necessarily mean that a small decrease in sedentary behaviour might not have an effect on health outcomes in older adults. This is illustrated by the findings of Gibbs et al. (2016), whose intervention aimed at reducing sedentary time of older adults resulted in statistically significant positive effects on physical function and quality of life while there was only a small (statistically non-significant) reduction in sedentary time [33]. It is also important to be aware that the responsiveness coefficients (GR, ES) can only be interpreted in a manner relative to each other, because there was no change/difference induced between the measurements [21,25]. Finally, the study sample consisted of older adults and therefore the findings may not be generalizable to other groups who may have different day to day patterns of sedentary behaviour. For future research it is recommended to repeat the analyses in different populations, using a larger sample size, and to expand the analyses to other taxons of the TASST framework as well.

### Conclusion

There is no single sedentary behaviour tool that is most appropriate to measure and interpret change in sedentary behaviour in older adults in every situation and type of study. To choose the most appropriate measure accuracy, reliability and responsiveness should be balanced in a way that suits the specific aim and design of the study or surveillance. Using objective measurements, in quasi-experimental, longitudinal and controlled trial study designs, a one day measurement is more responsive to change than an average day in a 7-day measurement. In contrast, in a clinical practice an average day in a 7-day measurement is more responsive to change than a one day measurement. The best subjective method is also context dependent, but asking about a single item proxy measure of watching TV over a previous week recall period, is the more reliable and responsive method in most situations. However, TV viewing may have limitations as a reliable measure of total sedentary time, as it may not pick up an exchange of time from TV viewing to other sedentary behaviours.

## Supporting information

S1 Appendix Daily SB questionnaire. (PDF)

S1 Data file (XLSX)

## References

[1] Biswas A, Oh PI, Faulkner GE, Bajaj RR, Silver MA, Mitchell MS, et al Sedentary time and its association with risk for disease incidence, mortality, and hospitalization in adults: a systematic review and meta-analysis. Ann Intern Med. 2015;162: 123–132. doi: 10.7326/M14-1651 25599350 10.7326/M14-1651

[2] Australian Government, Department of Health. Australia's physical activity and sedentary behaviour guidelines. Adults. 2014.

[3] Department of Health, Physical Activity, Health Improvement and Protection. Start Active, Stay Active: A report on physical activity from the four home countries’ Chief Medical Officers. 2011.

[4] Martin A, Fitzsimons C, Jepson R, Saunders DH, van der Ploeg HP, Teixeira PJ, et al Interventions with potential to reduce sedentary time in adults: systematic review and meta-analysis. British Journal of Sports Medicine. 2015. 10.1136/bjsports-2014-094524 25907181

[5] Fitzsimons CF, Kirk A, Baker G, Michie F, Kane C, Mutrie N. Using an individualised consultation and activPAL feedback to reduce sedentary time in older Scottish adults: results of a feasibility and pilot study. Prev Med. 2013;57: 718–720. doi: 10.1016/j.ypmed.2013.07.017 23891853 10.1016/j.ypmed.2013.07.017

[6] Gardiner PA, Eakin EG, Healy GN, Owen N. Feasibility of reducing older adults' sedentary time. Am J Prev Med. 2011;41: 174–177. doi: 10.1016/j.amepre.2011.03.020 21767725 10.1016/j.amepre.2011.03.020

[7] Hart TL, Swartz AM, Cashin SE, Strath SJ. How many days of monitoring predict physical activity and sedentary behaviour in older adults? Int J Behav Nutr Phys Act. 2011;8: 62-5868-8-62. 10.1186/1479-5868-8-62 PMC3130631 21679426

[8] Pedersen ES, Danquah IH, Petersen CB, Tolstrup JS. Intra-individual variability in day-to-day and month-to-month measurements of physical activity and sedentary behaviour at work and in leisure-time among Danish adults. BMC Public Health. 2016;16: 1222 doi: 10.1186/s12889-016-3890-3 27914468 10.1186/s12889-016-3890-3 PMC5135790

[9] Dall PM, Coulter EH, Fitzsimons CF, Skelton DA, Chastin SFM. The TAxonomy of Self-reported Sedentary behaviour Tools (TASST) framework for development, comparison and evaluation of self-report tools: content analysis and systematic review. 2017;7: e013844. 10.1136/bmjopen-2016-013844 PMC5775464 28391233

[10] O'Connell SE, Griffiths PL, Clemes SA. Seasonal variation in physical activity, sedentary behaviour and sleep in a sample of UK adults. Ann Hum Biol. 2014;41: 1–8. doi: 10.3109/03014460.2013.827737 23992280 10.3109/03014460.2013.827737

[11] Beaton DE, Bombardier C, Katz JN, Wright JG. A taxonomy for responsiveness. J Clin Epidemiol. 2001;54: 1204–1217. 11750189 10.1016/s0895-4356(01)00407-3

[12] Kozey-Keadle S, Libertine A, Lyden K, Staudenmayer J, Freedson PS. Validation of Wearable Monitors for Assessing Sedentary Behavior. Med Sci Sports Exerc. 2011;43: 1561–1567. doi: 10.1249/MSS.0b013e31820ce174 21233777 10.1249/MSS.0b013e31820ce174

[13] Grant PM, Ryan CG, Tigbe WW, Granat MH. The validation of a novel activity monitor in the measurement of posture and motion during everyday activities. Br J Sports Med. 2006;40: 992–997. doi: 10.1136/bjsm.2006.030262 16980531 10.1136/bjsm.2006.030262 PMC2577473

[14] Grant PM, Dall PM, Mitchell SL, Granat MH. Activity-Monitor Accuracy in Measuring Step Number and Cadence in Community-Dwelling Older Adults. 2008;16: 201–214. 10.1123/japa.16.2.201 18483442

[15] Sellers C, Dall P, Grant M, Stansfield B. Validity and reliability of the activPAL3 for measuring posture and stepping in adults and young people. Gait Posture. 2016;43: 42–47. doi: 10.1016/j.gaitpost.2015.10.020 26669950 10.1016/j.gaitpost.2015.10.020

[16] R Development Core Team. R: A language and environment for statistical computing. 2011.

[17] Weir JP. Quantifying test-retest reliability using the intraclass correlation coefficient and the SEM. J Strength Cond Res. 2005;19: 231–240. doi: 10.1519/15184.1 15705040 10.1519/15184.1

[18] Overend T, Anderson C, Sawant A, Perryman B, Locking-Cusolito H. Relative and absolute reliability of physical function measures in people with end-stage renal disease. Physiother Can. 2010;62: 122–128. doi: 10.3138/physio.62.2.122 21359043 10.3138/physio.62.2.122 PMC2871020

[19] Harvill LM. Standard Error of Measurement. Educational Measurement: Issues and Practice. 1991;2(10): 33–41.

[20] Ries JD, Echternach JL, Nof L, Gagnon Blodgett M. Test-retest reliability and minimal detectable change scores for the timed "up & go" test, the six-minute walk test, and gait speed in people with Alzheimer disease. Phys Ther. 2009;89: 569–579. doi: 10.2522/ptj.20080258 19389792 10.2522/ptj.20080258

[21] Norman GR, Wyrwich KW, Patrick DL. The mathematical relationship among different forms of responsiveness coefficients. Qual Life Res. 2007;16: 815–822. doi: 10.1007/s11136-007-9180-x 17351823 10.1007/s11136-007-9180-x

[22] Beckerman H, Roebroeck ME, Lankhorst GJ, Becher JG, Bezemer PD, Verbeek AL. Smallest real difference, a link between reproducibility and responsiveness. Qual Life Res. 2001;10: 571–578. 11822790 10.1023/a:1013138911638

[23] Norman GR, Stratford P, Regehr G. Methodological problems in the retrospective computation of responsiveness to change: the lesson of Cronbach. J Clin Epidemiol. 1997;50: 869–879. 9291871 10.1016/s0895-4356(97)00097-8

[24] Stucki G, Liang MH, Fossel AH, Katz JN. Relative responsiveness of condition-specific and generic health status measures in degenerative lumbar spinal stenosis. J Clin Epidemiol. 1995;48: 1369–1378. 7490600 10.1016/0895-4356(95)00054-2

[25] Husted JA, Cook RJ, Farewell VT, Gladman DD. Methods for assessing responsiveness: a critical review and recommendations. J Clin Epidemiol. 2000;53: 459–468. 10812317 10.1016/s0895-4356(99)00206-1

[26] Kelly P, Fitzsimons C, Baker G. Should we reframe how we think about physical activity and sedentary behaviour measurement? Validity and reliability reconsidered. Int J Behav Nutr Phys Act. 2016;13: 32-016-0351-4. 10.1186/s12966-016-0351-4 PMC4772314 26931142

[27] Aadland E, Ylvisaker E. Reliability of Objectively Measured Sedentary Time and Physical Activity in Adults. PLoS One. 2015;10: e0133296 doi: 10.1371/journal.pone.0133296 26192184 10.1371/journal.pone.0133296 PMC4508000

[28] Donaldson SC, Montoye AH, Tuttle MS, Kaminsky LA. Variability of Objectively Measured Sedentary Behavior. Med Sci Sports Exerc. 2016;48: 755–761. doi: 10.1249/MSS.0000000000000828 26606270 10.1249/MSS.0000000000000828

[29] Kolotkin RL, Crosby RD, Williams GR, Hartley GG, Nicol S. The relationship between health-related quality of life and weight loss. Obes Res. 2001;9: 564–571. doi: 10.1038/oby.2001.73 11557837 10.1038/oby.2001.73

[30] Nunnally JC, Bernstein IH. Psychometric Theory. New York, NY: McGraw Hill; 1994.

[31] Copay AG, Subach BR, Glassman SD, Polly DW Jr, Schuler TC. Understanding the minimum clinically important difference: a review of concepts and methods. Spine J. 2007;7: 541–546. doi: 10.1016/j.spinee.2007.01.008 17448732 10.1016/j.spinee.2007.01.008

[32] de Vet HC, Terwee CB, Knol DL, Bouter LM. When to use agreement versus reliability measures. J Clin Epidemiol. 2006;59: 1033–1039. doi: 10.1016/j.jclinepi.2005.10.015 16980142 10.1016/j.jclinepi.2005.10.015

[33] Barone Gibbs B, Brach JS, Byard T, Creasy S, Davis KK, McCoy S, et al Reducing Sedentary Behavior Versus Increasing Moderate-to-Vigorous Intensity Physical Activity in Older Adults: A 12-Week Randomized, Clinical Trial. J Aging Health. 2016. 10.1177/0898264316635564 26944808

