# Supplementary material for: Reliability, minimal detectable change and responsiveness to change: Indicators to select the best method to measure sedentary behaviour in older adults in different study designs
Source: PLoS One. 2018 Apr 12;13(4):e0195424. doi: 10.1371/journal.pone.0195424 (PMC5896945; doi:10.1371/journal.pone.0195424)
Supplement: S1 Appendix — (PDF) [file pone.0195424.s001.pdf]

GCU Glasgow Caledonian University

SENIORS USP
UNDERSTANDING SEDENTARY PATTERNS

Daily questionnaire

“Dynamics of sedentary behavior in older adults “

A study researcher can be contacted during office hours on: xxxx

Office use only

Participant ID:

Start date:

Researcher:

INSTRUCTIONS

Daily questionnaire

This booklet starts with Day 1 and finishes with Day 15.

Please read carefully, follow the instructions, and try to answer the questions as well as you can.
We realise that some questions may be difficult to answer, but try to give your best estimate or guess.

You are wearing an activity monitor on your leg. If you experience any skin irritation, please stop wearing the monitor and let us know.

A study researcher can be contacted during office hours on: xxxx.

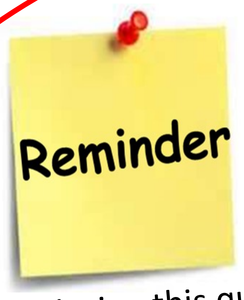

Try placing this questionnaire somewhere that you always go first thing in the morning, such as next to the kettle or with your pills, to help you remember to fill it in at 9.00 am.

Participant ID:

INSTRUCTIONS

Daily questionnaire

In order that we can understand the monitor readings it is very important for us to know:

The time you go to bed each night

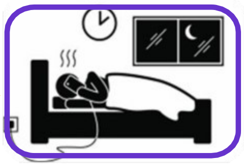

And

The time you get up each day

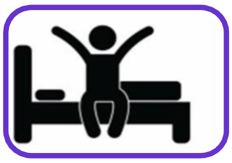

We would also like to know your best guess of:

The time you fell asleep each night

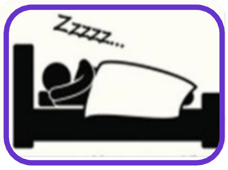

And

The time you woke up each day

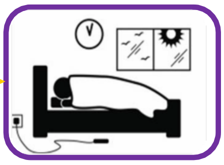

Even if you don't know exactly what time you fell asleep or woke up, please guess as best you can.

Example

If you went to bed at 10:17 in the evening, but think you went to sleep at 12:45 in the morning, you would write:

				Please circle
1. What time did you get into bed last night?	10	17	am	pm (circled)
2. What time do you think you fell asleep last night?	12	45	am (circled)	pm

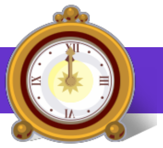

Remember that midnight, and times after midnight, are a.m.

Participant ID:

Sun/Mon/Tues/Wed/Thurs/Fri/Sat - - 2015

DAY 1

Daily questionnaire

Morning: Please answer the following questions around 9.00 am

Please circle

**1.**	**What time did you get into bed last night?**	____ : ____	am pm
**2.**	**What time do you think you fell asleep last night?**	____ : ____	am pm
**3.**	**How many times did you get out of bed during the night last night?**	____	times
**4.**	**What time did you wake up this morning?**	____ : ____	am pm
**5.**	**What time did you get out of bed this morning?**	____ : ____	am pm

Participant ID:

Sun/Mon/Tues/Wed/Thurs/Fri/Sat - - 2015

DAY 2

Daily questionnaire

Morning: Please answer the following questions around 9.00 am

Please circle

**1.**	**What time did you get into bed last night?**		:		am pm
**2.**	**What time do you think you fell asleep last night?**		:		am pm
**3.**	**How many times did you get out of bed during the night last night?**				times
**4.**	**What time did you wake up this morning?**		:		am pm
**5.**	**What time did you get out of bed this morning?**		:		am pm

Participant ID:

Thank you for today. Please continue tomorrow.

Sun/Mon/Tues/Wed/Thurs/Fri/Sat - - 2015

DAY 3

Daily questionnaire

Morning: Please answer the following questions around 9.00 am

Please circle

**1.**	**What time did you get into bed last night?**	:	am pm
**2.**	**What time do you think you fell asleep last night?**	:	am pm
**3.**	**How many times did you get out of bed during the night last night?**		times
**4.**	**What time did you wake up this morning?**	:	am pm
**5.**	**What time did you get out of bed this morning?**	:	am pm

Participant ID:

Sun/Mon/Tues/Wed/Thurs/Fri/Sat - - 2015

DAY 4

Daily questionnaire

Morning: Please answer the following questions around 9.00 am

Please circle

**1.**	**What time did you get into bed last night?**	:		am pm
**2.**	**What time do you think you fell asleep last night?**	:		am pm
**3.**	**How many times did you get out of bed during the night last night?**			times
**4.**	**What time did you wake up this morning?**	:		am pm
**5.**	**What time did you get out of bed this morning?**	:		am pm

Participant ID:

Thank you for today. Please continue tomorrow.

Sun/Mon/Tues/Wed/Thurs/Fri/Sat - - 2015

DAY 5

Daily questionnaire

Morning: Please answer the following questions around 9.00 am

Please circle

1. **What time did you get into bed last night?** ___ : ___ am pm
2. **What time do you think you fell asleep last night?** ___ : ___ am pm
3. **How many times did you get out of bed during the night last night?** ___ times
4. **What time did you wake up this morning?** ___ : ___ am pm
5. **What time did you get out of bed this morning?** ___ : ___ am pm

Participant ID:

Sun/Mon/Tues/Wed/Thurs/Fri/Sat - - 2015

DAY 5

Daily questionnaire

Sitting

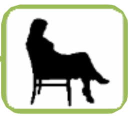

Reclining

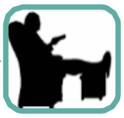

Lying down

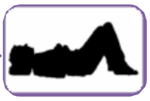

Time spent sitting yesterday

These questions are about what you did yesterday. We are interested in your sedentary behavior, which is any time you spend sitting, reclining and lying down. Please don't count the time asleep at night.

1. **Please estimate how long in total you spent sitting yesterday?**

(Please write in) ☐ hours ☐ minutes

The next questions are about what you were doing when you were sitting down yesterday.

We know that people sometimes do two things at once when sitting down, for example you might knit and listen to music, or eat and watch TV. In this case, please decide which one is the main activity and write in the time for that.

It may be difficult sometimes to decide which is the main activity, but please choose the one you think fits best.

Please don't worry about how these numbers add up. We know that this doesn't cover everything.

Participant ID:

Sun/Mon/Tues/Wed/Thurs/Fri/Sat - - 2015

DAY 5

Daily questionnaire

Example

If you had the TV on in the background while eating a meal, then eating is your main activity and you should write the time in that section. But if you were having a snack while watching your favourite TV show, then watching TV is your main activity.

2. **Please write in how much time you spent sitting and doing the following activities <u>yesterday</u>:**

		Hours	Minutes
a.	Watching television, video, or DVDs		
b.	Using a computer for work<br>(e.g., paid work, volunteering, clerical tasks)		
c.	Using a computer for leisure<br>(e.g., games, friends and family, internet, shopping)		
d.	Read for leisure<br>(e.g., books, newspaper, magazine, kindle)		
e.	Listening to music or radio		
f.	Doing a hobby<br>(e.g., knitting, crafts, jigsaws, puzzles, playing piano)		
g.	Talking with friends or family<br>(e.g., talking in person or on the phone)		
h.	Eating meals or snacks		
i.	Performing self-care tasks<br>(e.g., bathing, using toilet, dressing, brushing hair)		
j.	Household tasks<br>(e.g., cleaning shoes, writing a list, ironing, folding)		
k.	Taking a nap during the day or resting while doing nothing else		

Participant ID:

Thank you for today. Please continue tomorrow.

Sun/Mon/Tues/Wed/Thurs/Fri/Sat - - 2015

DAY 6

Daily questionnaire

Morning: Please answer the following questions around 9.00 am

Please circle

1. **What time did you get into bed last night?** ___ : ___ am pm
2. **What time do you think you fell asleep last night?** ___ : ___ am pm
3. **How many times did you get out of bed during the night last night?** ___ times
4. **What time did you wake up this morning?** ___ : ___ am pm
5. **What time did you get out of bed this morning?** ___ : ___ am pm

DAY 6

Daily questionnaire

Sitting

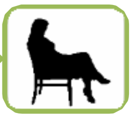

Reclining

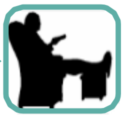

Lying down

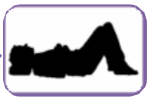

Time spent sitting yesterday

These questions are about what you did yesterday. We are interested in your sedentary behavior, which is any time you spend sitting, reclining and lying down. Please don't count the time asleep at night.

1. **Please estimate how long in total you spent sitting yesterday?**

(Please write in) ☐ hours ☐ minutes

The next questions are about what you were doing when you were sitting down yesterday.

We know that people sometimes do two things at once when sitting down, for example you might knit and listen to music, or eat and watch TV. In this case, please decide which one is the main activity and write in the time for that.

It may be difficult sometimes to decide which is the main activity, but please choose the one you think fits best.

Please don't worry about how these numbers add up. We know that this doesn't cover everything.

Sun/Mon/Tues/Wed/Thurs/Fri/Sat - - 2015

DAY 6

Daily questionnaire

Example

If you had the TV on in the background while eating a meal, then eating is your main activity and you should write the time in that section. But if you were having a snack while watching your favourite TV show, then watching TV is your main activity.

2. **Please write in how much time you spent sitting and doing the following activities yesterday:**

	Activity	Hours	Minutes
a.	Watching television, video, or DVDs		
b.	Using a computer for work (e.g., paid work, volunteering, clerical tasks)		
c.	Using a computer for leisure (e.g., games, friends and family, internet, shopping)		
d.	Read for leisure (e.g., books, newspaper, magazine, kindle)		
e.	Listening to music or radio		
f.	Doing a hobby (e.g., knitting, crafts, jigsaws, puzzles, playing piano)		
g.	Talking with friends or family (e.g., talking in person or on the phone)		
h.	Eating meals or snacks		
i.	Performing self-care tasks (e.g., bathing, using toilet, dressing, brushing hair)		
j.	Household tasks (e.g., cleaning shoes, writing a list, ironing, folding)		
k.	Taking a nap during the day or resting while doing nothing else		

Participant ID:

Thank you for today. Please continue tomorrow.

Sun/Mon/Tues/Wed/Thurs/Fri/Sat - - 2015

DAY 7

Daily questionnaire

Morning: Please answer the following questions around 9.00 am

Please circle

1. **What time did you get into bed last night?** ___ : ___ am pm
2. **What time do you think you fell asleep last night?** ___ : ___ am pm
3. **How many times did you get out of bed during the night last night?** ___ times
4. **What time did you wake up this morning?** ___ : ___ am pm
5. **What time did you get out of bed this morning?** ___ : ___ am pm

Sun/Mon/Tues/Wed/Thurs/Fri/Sat - - 2015

DAY 7

Daily questionnaire

Sitting

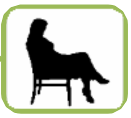

Reclining

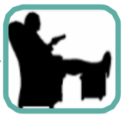

Lying down

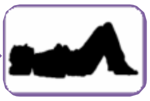

Time spent sitting yesterday

These questions are about what you did yesterday. We are interested in your sedentary behavior, which is any time you spend sitting, reclining and lying down. Please don't count the time asleep at night.

1. **Please estimate how long in total you spent sitting yesterday?**

   (Please write in) ☐ hours ☐ minutes

The next questions are about what you were doing when you were sitting down yesterday.

We know that people sometimes do two things at once when sitting down, for example you might knit and listen to music, or eat and watch TV. In this case, please decide which one is the main activity and write in the time for that.

It may be difficult sometimes to decide which is the main activity, but please choose the one you think fits best.

Please don't worry about how these numbers add up. We know that this doesn't cover everything.

Participant ID:

Sun/Mon/Tues/Wed/Thurs/Fri/Sat - - 2015

DAY 7

Daily questionnaire

Example

If you had the TV on in the background while eating a meal, then eating is your main activity and you should write the time in that section. But if you were having a snack while watching your favourite TV show, then watching TV is your main activity.

2. **Please write in how much time you spent sitting and doing the following activities <u>yesterday</u>:**

		Hours	Minutes
a.	Watching television, video, or DVDs		
b.	Using a computer for work<br>(e.g., paid work, volunteering, clerical tasks)		
c.	Using a computer for leisure<br>(e.g., games, friends and family, internet, shopping)		
d.	Read for leisure<br>(e.g., books, newspaper, magazine, kindle)		
e.	Listening to music or radio		
f.	Doing a hobby<br>(e.g., knitting, crafts, jigsaws, puzzles, playing piano)		
g.	Talking with friends or family<br>(e.g., talking in person or on the phone)		
h.	Eating meals or snacks		
i.	Performing self-care tasks<br>(e.g., bathing, using toilet, dressing, brushing hair)		
j.	Household tasks<br>(e.g., cleaning shoes, writing a list, ironing, folding)		
k.	Taking a nap during the day or resting while doing nothing else		

Participant ID:

Thank you for today. Please continue tomorrow.

Sun/Mon/Tues/Wed/Thurs/Fri/Sat - - 2015

DAY 8

Daily questionnaire

Morning: Please answer the following questions around 9.00 am

				Please circle
1.	What time did you get into bed last night?	:		am pm
2.	What time do you think you fell asleep last night?	:		am pm
3.	How many times did you get out of bed during the night last night?			times
4.	What time did you wake up this morning?	:		am pm
5.	What time did you get out of bed this morning?	:		am pm

DAY 8

Daily questionnaire

Sitting

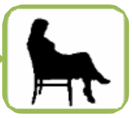

Reclining

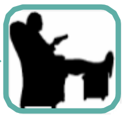

Lying down

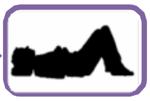

Time spent sitting yesterday

These questions are about what you did yesterday. We are interested in your sedentary behavior, which is any time you spend sitting, reclining and lying down. Please don't count the time asleep at night.

1. **Please estimate how long in total you spent sitting yesterday?**

   (Please write in) ☐ hours ☐ minutes

The next questions are about what you were doing when you were sitting down yesterday.

We know that people sometimes do two things at once when sitting down, for example you might knit and listen to music, or eat and watch TV. In this case, please decide which one is the main activity and write in the time for that.

It may be difficult sometimes to decide which is the main activity, but please choose the one you think fits best.

Please don't worry about how these numbers add up. We know that this doesn't cover everything.

Sun/Mon/Tues/Wed/Thurs/Fri/Sat - - 2015

DAY 8

Daily questionnaire

Example

If you had the TV on in the background while eating a meal, then eating is your main activity and you should write the time in that section. But if you were having a snack while watching your favourite TV show, then watching TV is your main activity.

2. **Please write in how much time you spent sitting and doing the following activities <u>yesterday</u>:**

		Hours	Minutes
a.	Watching television, video, or DVDs		
b.	Using a computer for work<br>(e.g., paid work, volunteering, clerical tasks)		
c.	Using a computer for leisure<br>(e.g., games, friends and family, internet, shopping)		
d.	Read for leisure<br>(e.g., books, newspaper, magazine, kindle)		
e.	Listening to music or radio		
f.	Doing a hobby<br>(e.g., knitting, crafts, jigsaws, puzzles, playing piano)		
g.	Talking with friends or family<br>(e.g., talking in person or on the phone)		
h.	Eating meals or snacks		
i.	Performing self-care tasks<br>(e.g., bathing, using toilet, dressing, brushing hair)		
j.	Household tasks<br>(e.g., cleaning shoes, writing a list, ironing, folding)		
k.	Taking a nap during the day or resting while doing nothing else		

Participant ID:

There are more questions today. Please continue on the next page.

DAY 8

Daily questionnaire

Sitting
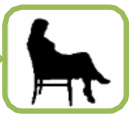

Reclining
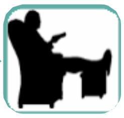

Lying down
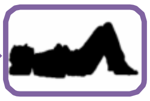

Time spent sitting on an average day in the last 7 days

The following questions are about the last seven days (week). Please answer them at some time today.

Please think about the time you spent sitting over the last 7 days.

We will be asking about the time you spent sitting over the last seven days.

We will be asking you to estimate the time you spent sitting on an average day in the last seven days. We realize this will vary over the week, but try to give an estimate or guess.

1. **Please estimate how long in total you spent sitting on an average day in the last seven days?**

(Please write in) hours minutes

We would now like to ask you some questions about what you were doing when you were sitting down over the last week.

It may be difficult sometimes to decide which is the main activity, but please choose the one you think fits best. Please don't worry about how these numbers add up. We know that this doesn't cov-er everything.

Sun/Mon/Tues/Wed/Thurs/Fri/Sat - - 2015

DAY 8

Daily questionnaire

Example

If you had the TV on in the background while eating a meal, then eating is your main activity and you should write the time in that section. But if you were having a snack while watching your favourite TV show, then watching TV is your main activity.

2. **Please write in how much time you spent sitting and doing the following activities <u>on an average day in the last seven days</u>**:

		Hours	Minutes
a.	Watching television, video, or DVDs		
b.	Using a computer for work (e.g., paid work, volunteering, clerical tasks)		
c.	Using a computer for leisure (e.g., games, friends and family, internet, shopping)		
d.	Read for leisure (e.g., books, newspaper, magazine, kindle)		
e.	Listening to music or radio		
f.	Doing a hobby (e.g., knitting, crafts, jigsaws, puzzles, playing piano)		
g.	Talking with friends or family (e.g., talking in person or on the phone)		
h.	Eating meals or snacks		
i.	Performing self-care tasks (e.g., bathing, using toilet, dressing, brushing hair)		
j.	Household tasks (e.g., cleaning shoes, writing a list, ironing, folding)		
k.	Taking a nap during the day or resting while doing nothing else		

Sun/Mon/Tues/Wed/Thurs/Fri/Sat ____ - ____ - 2015

DAY 9

Daily questionnaire

Morning: Please answer the following questions around 9.00 am

Please circle

1. **What time did you get into bed last night?** ____ : ____ am pm
2. **What time do you think you fell asleep last night?** ____ : ____ am pm
3. **How many times did you get out of bed during the night last night?** ____ times
4. **What time did you wake up this morning?** ____ : ____ am pm
5. **What time did you get out of bed this morning?** ____ : ____ am pm

DAY 9

Daily questionnaire

Sitting

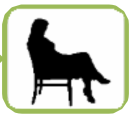

Reclining

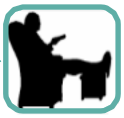

Lying down

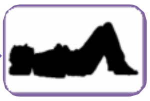

Time spent sitting yesterday

These questions are about what you did yesterday. We are interested in your sedentary behavior, which is any time you spend sitting, reclining and lying down. Please don't count the time asleep at night.

1. **Please estimate how long in total you spent sitting yesterday?**

(Please write in) ☐ hours ☐ minutes

The next questions are about what you were doing when you were sitting down yesterday.

We know that people sometimes do two things at once when sitting down, for example you might knit and listen to music, or eat and watch TV. In this case, please decide which one is the main activity and write in the time for that.

It may be difficult sometimes to decide which is the main activity, but please choose the one you think fits best.

Please don't worry about how these numbers add up. We know that this doesn't cover everything.

DAY 9

Daily questionnaire

Example

If you had the TV on in the background while eating a meal, then eating is your main activity and you should write the time in that section. But if you were having a snack while watching your favourite TV show, then watching TV is your main activity.

2. **Please write in how much time you spent sitting and doing the following activities <u>yesterday</u>:**

		Hours	Minutes
a.	Watching television, video, or DVDs		
b.	Using a computer for work (e.g., paid work, volunteering, clerical tasks)		
c.	Using a computer for leisure (e.g., games, friends and family, internet, shopping)		
d.	Read for leisure (e.g., books, newspaper, magazine, kindle)		
e.	Listening to music or radio		
f.	Doing a hobby (e.g., knitting, crafts, jigsaws, puzzles, playing piano)		
g.	Talking with friends or family (e.g., talking in person or on the phone)		
h.	Eating meals or snacks		
i.	Performing self-care tasks (e.g., bathing, using toilet, dressing, brushing hair)		
j.	Household tasks (e.g., cleaning shoes, writing a list, ironing, folding)		
k.	Taking a nap during the day or resting while doing nothing else		

Participant ID:

Thank you for today. Please continue tomorrow.

Sun/Mon/Tues/Wed/Thurs/Fri/Sat - - 2015

DAY 10

Daily questionnaire

Morning: Please answer the following questions around 9.00 am

Please circle

**1.**	**What time did you get into bed last night?**	:	am pm
**2.**	**What time do you think you fell asleep last night?**	:	am pm
**3.**	**How many times did you get out of bed during the night last night?**		times
**4.**	**What time did you wake up this morning?**	:	am pm
**5.**	**What time did you get out of bed this morning?**	:	am pm

Sun/Mon/Tues/Wed/Thurs/Fri/Sat - - 2015

DAY 10

Daily questionnaire

Sitting 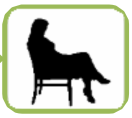

Reclining 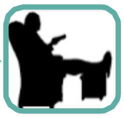

Lying down 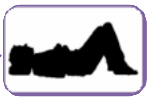

Time spent sitting yesterday

These questions are about what you did yesterday. We are interested in your sedentary behavior, which is any time you spend sitting, reclining and lying down. Please don't count the time asleep at night.

1. **Please estimate how long in total you spent sitting yesterday?**

(Please write in) ☐ hours ☐ minutes

The next questions are about what you were doing when you were sitting down yesterday.

We know that people sometimes do two things at once when sitting down, for example you might knit and listen to music, or eat and watch TV. In this case, please decide which one is the main activity and write in the time for that.

It may be difficult sometimes to decide which is the main activity, but please choose the one you think fits best.

Please don't worry about how these numbers add up. We know that this doesn't cover everything.

Participant ID:

Sun/Mon/Tues/Wed/Thurs/Fri/Sat - - 2015

DAY 10

Daily questionnaire

Example

If you had the TV on in the background while eating a meal, then eating is your main activity and you should write the time in that section. But if you were having a snack while watching your favourite TV show, then watching TV is your main activity.

2. **Please write in how much time you spent sitting and doing the following activities <u>yesterday</u>:**

		Hours	Minutes
a.	Watching television, video, or DVDs		
b.	Using a computer for work<br>(e.g., paid work, volunteering, clerical tasks)		
c.	Using a computer for leisure<br>(e.g., games, friends and family, internet, shopping)		
d.	Read for leisure<br>(e.g., books, newspaper, magazine, kindle)		
e.	Listening to music or radio		
f.	Doing a hobby<br>(e.g., knitting, crafts, jigsaws, puzzles, playing piano)		
g.	Talking with friends or family<br>(e.g., talking in person or on the phone)		
h.	Eating meals or snacks		
i.	Performing self-care tasks<br>(e.g., bathing, using toilet, dressing, brushing hair)		
j.	Household tasks<br>(e.g., cleaning shoes, writing a list, ironing, folding)		
k.	Taking a nap during the day or resting while doing nothing else		

Participant ID:

Thank you for today. Please continue tomorrow.

Sun/Mon/Tues/Wed/Thurs/Fri/Sat - - 2015

DAY 11

Daily questionnaire

Morning: Please answer the following questions around 9.00 am

Please circle

**1.**	**What time did you get into bed last night?**	:		am pm
**2.**	**What time do you think you fell asleep last night?**	:		am pm
**3.**	**How many times did you get out of bed during the night last night?**			times
**4.**	**What time did you wake up this morning?**	:		am pm
**5.**	**What time did you get out of bed this morning?**	:		am pm

DAY 11

Daily questionnaire

Sitting
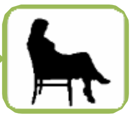

Reclining
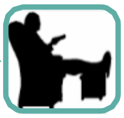

Lying down
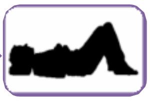

Time spent sitting yesterday

These questions are about what you did yesterday. We are interested in your sedentary behavior, which is any time you spend sitting, reclining and lying down. Please don't count the time asleep at night.

1. **Please estimate how long in total you spent sitting yesterday?**

(Please write in) ☐ hours ☐ minutes

The next questions are about what you were doing when you were sitting down yesterday.

We know that people sometimes do two things at once when sitting down, for example you might knit and listen to music, or eat and watch TV. In this case, please decide which one is the main activity and write in the time for that.

It may be difficult sometimes to decide which is the main activity, but please choose the one you think fits best.

Please don't worry about how these numbers add up. We know that this doesn't cover everything.

Sun/Mon/Tues/Wed/Thurs/Fri/Sat - - 2015

DAY 11

Daily questionnaire

Example

If you had the TV on in the background while eating a meal, then eating is your main activity and you should write the time in that section. But if you were having a snack while watching your favourite TV show, then watching TV is your main activity.

2. **Please write in how much time you spent sitting and doing the following activities <u>yesterday</u>:**

		Hours	Minutes
a.	Watching television, video, or DVDs		
b.	Using a computer for work (e.g., paid work, volunteering, clerical tasks)		
c.	Using a computer for leisure (e.g., games, friends and family, internet, shopping)		
d.	Read for leisure (e.g., books, newspaper, magazine, kindle)		
e.	Listening to music or radio		
f.	Doing a hobby (e.g., knitting, crafts, jigsaws, puzzles, playing piano)		
g.	Talking with friends or family (e.g., talking in person or on the phone)		
h.	Eating meals or snacks		
i.	Performing self-care tasks (e.g., bathing, using toilet, dressing, brushing hair)		
j.	Household tasks (e.g., cleaning shoes, writing a list, ironing, folding)		
k.	Taking a nap during the day or resting while doing nothing else		

Participant ID:

Thank you for today. Please continue tomorrow.

Sun/Mon/Tues/Wed/Thurs/Fri/Sat - - 2015

DAY 12

Daily questionnaire

Morning: Please answer the following questions around 9.00 am

Please circle

1.	**What time did you get into bed last night?**	:		am pm
2.	**What time do you think you fell asleep last night?**	:		am pm
3.	**How many times did you get out of bed during the night last night?**			times
4.	**What time did you wake up this morning?**	:		am pm
5.	**What time did you get out of bed this morning?**	:		am pm

Sun/Mon/Tues/Wed/Thurs/Fri/Sat - - 2015

DAY 13

Daily questionnaire

Morning: Please answer the following questions around 9.00 am

Please circle

**1.**	**What time did you get into bed last night?**		:		am pm
**2.**	**What time do you think you fell asleep last night?**		:		am pm
**3.**	**How many times did you get out of bed during the night last night?**				times
**4.**	**What time did you wake up this morning?**		:		am pm
**5.**	**What time did you get out of bed this morning?**		:		am pm

Participant ID:

DAY 14

Daily questionnaire

Morning: Please answer the following questions around 9.00 am

		Please circle
1. What time did you get into bed last night?	:	am pm
2. What time do you think you fell asleep last night?	:	am pm
3. How many times did you get out of bed during the night last night?		times
4. What time did you wake up this morning?	:	am pm
5. What time did you get out of bed this morning?	:	am pm

Participant ID:

Thank you for today. Please continue tomorrow.

Sun/Mon/Tues/Wed/Thurs/Fri/Sat - - 2015

DAY 15

Daily questionnaire

Morning: Please answer the following questions around 9.00 am

Please circle

1. **What time did you get into bed last night?** ___ : ___ am pm
2. **What time do you think you fell asleep last night?** ___ : ___ am pm
3. **How many times did you get out of bed during the night last night?** ___ times
4. **What time did you wake up this morning?** ___ : ___ am pm
5. **What time did you get out of bed this morning?** ___ : ___ am pm

Sitting

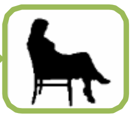

Reclining

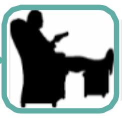

Lying down

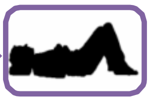

Time spent sitting on an average day in the last 7 days

The following questions are about the last seven days (week). Please answer them at some time today.

Please think about the time you spent sitting over the last 7 days.

1. **Please estimate how long in total you spent sitting on an average day in the last seven days?**

(Please write in) ___ hours ___ minutes

Participant ID:

DAY 15

Daily questionnaire

We would now like to ask you some questions about what you were doing when you were sitting down over the last week.

We know that people sometimes do two things at once when sitting down, for example you might knit and listen to music, or eat and watch TV. In this case, please decide which one is the main activity and write in the time for that.

It may be difficult sometimes to decide which is the main activity, but please choose the one you think fits best.

Please don't worry about how these numbers add up. We know that this doesn't cover everything.

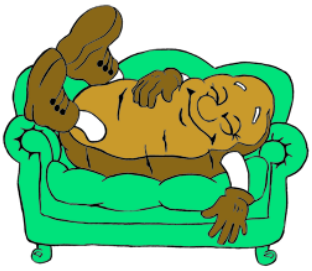

Example

If you had the TV on in the background while eating a meal, then eating is your main activity and you should write the time in that section. But if you were having a snack while watching your favourite TV show, then watching TV is your main activity.

Participant ID:

There are more questions today. Please continue on the next page.

Sun/Mon/Tues/Wed/Thurs/Fri/Sat - - 2015

DAY 15

Daily questionnaire

2. **Please write in how much time you spent sitting and doing the following activities <u>on an average day in the last seven days</u>**:

		Hours	Minutes
a.	Watching television, video, or DVDs		
b.	Using a computer for work (e.g., paid work, volunteering, clerical tasks)		
c.	Using a computer for leisure (e.g., games, friends and family, internet, shopping)		
d.	Read for leisure (e.g., books, newspaper, magazine, kindle)		
e.	Listening to music or radio		
f.	Doing a hobby (e.g., knitting, crafts, jigsaws, puzzles, playing piano)		
g.	Talking with friends or family (e.g., talking in person or on the phone)		
h.	Eating meals or snacks		
i.	Performing self-care tasks (e.g., bathing, using toilet, dressing, brushing hair)		
j.	Household tasks (e.g., cleaning shoes, writing a list, ironing, folding)		
k.	Taking a nap during the day or resting while doing nothing else		

Thank you for taking the time to complete this questionnaire.

Please return this questionnaire to the researcher when you see him/her at your next visit.
